# Prevalence of human T-lymphotropic virus type 1 and 2 (HTLV-1/-2) infection in pregnant women in Brazil: a systematic review and meta-analysis

**DOI:** 10.1038/s41598-021-94934-7

**Published:** 2021-07-28

**Authors:** Bruna Angelo Vieira, Augusto Bacelo Bidinotto, William Jones Dartora, Luana Giongo Pedrotti, Vanessa Martins de Oliveira, Eliana Márcia Wendland

**Affiliations:** 1 Hospital Moinhos de Vento, Porto Alegre, Brazil; 2 Public Health Department, Federal University of Health Sciences of Porto Alegre, Porto Alegre, Brazil

**Keywords:** Public health, Epidemiology, Population screening

## Abstract

Human T-lymphotropic virus type 1 (HTLV-1) infection may cause serious disease, while pathogenicity of HTLV-2 is less certain. There are no screening or surveillance programs for HTLV-1/-2 infection in Brazil. By performing this systematic review, we aimed to estimate the prevalence of HTLV-1/-2 infections in pregnant women in Brazil. This review included cohort and cross-sectional studies that assessed the presence of either HTLV-1/-2 infection in pregnant women in Brazil. We searched BVS/LILACS, Cochrane Library/CENTRAL, EMBASE, PubMed/MEDLINE, Scopus, Web of Science and gray literature from inception to August 2020. We identified 246 records in total. Twenty-six of those were included in the qualitative synthesis, while 17 of them were included in the meta-analysis. The prevalence of HTLV-1 in Brazilian pregnant women, as diagnosed by a positive screening test and a subsequent positive confirmatory test, was 0.32% (95% CI 0.19–1.54), while of HTLV-2 was 0.04% (95% CI 0.02–0.08). Subgroup analysis by region showed the highest prevalence in the Northeast region (0.60%; 95% CI 0.37–0.97) for HTLV-1 and in the South region (0.16%; 95% CI 0.02–1.10) for HTLV-2. The prevalence of HTLV-1 is much higher than HTLV-2 infection in pregnant Brazilian women with important differences between regions. The prevalence of both HTLV-1/-2 are higher in the Northeast compared to Center-West region.

## Introduction

The human T-lymphotropic virus type 1 (HTLV-1), also known as human T-cell leukemia virus, was the first human retrovirus to be discovered and isolated^1^, and over the years other human T-lymphotropic viruses subtypes have been discovered^2–4^. It is mainly transmitted through sexual contact, sharing of syringes and needles, blood transfusion and vertical transmission from mother to child through breastfeeding^5^. The virus geographical distribution follows a clustering pattern—areas with a high prevalence of endemic infection are adjacent to areas in which the disease is extremely rare^6^. While prevalence seems to be decreasing in endemic areas^7^, such as in Japan that has several government measures and actions have been implemented to prevent new HTLV-1 infection^8^, it remains stable in areas in which infection rates are relatively low^9^.

HTLV-1 infection leads to serious disease in approximately 10% of infected individuals^10^. Adult T-cell leukemia (ATL) and HTLV-1-associated myelopathy/tropical spastic paraparesis (HAM/TSP) are the conditions most commonly associated with HTLV-1 infection; while development of these conditions is relatively rare, their unfavorable prognosis makes them particularly important. In addition to these major diseases, HTLV-1 infection has also been associated with uveitis and dermatological lesions, and the appearance of these conditions may indicate the future development of ATL or HAM/TSP^10,11^. A recent meta-analysis showed that HTLV-1 is associated with increased all-cause mortality, inflammatory conditions (eg, fibromyalgia), infectious conditions (eg Strongyloides stercoralis hyperinfection syndrome and tuberculosis), and other types of cancer (eg Lymphoma other than ATL)^12^.

The HTLV-2 is considered an ancestral infection and this subtype can be used as a marker of human migration^13^. The pathogenicity of HTLV-2 is less certain, also has been linked to HAM/TSP, besides a connection to other neurological and pulmonary disorders^14^. On the other hand, HTLV-3 and HTLV-4, were more recently described in isolated forest areas of Cameroon^3,4^, and not yet associated with clinical manifestations^15,16^.

Despite significant morbidity and mortality in some cases, persons with HTLV-1 infection are otherwise mostly asymptomatic^5,17^. This makes cases hard to detect and creates silent networks of transmission in restricted areas with high endemicity, contributing to the HTLV-1 distribution in somewhat well-delimited clusters. Its main modes of transmission tend to vary by study area; sexual transmission is predominant in some areas^18^, while some authors report that transmission is common through breastfeeding^19–21^.

Although HTLV-1/-2 during pregnancy is within the scope of surveillance program in Brazil, the screening test for HTLV-1/-2 among pregnant women is not implemented as a universal screening program in the Public Health System until now^22,23^. Also, the HTLV-1/-2 infection is not included in the list of conditions that require compulsory notification to the Brazilian Ministry of Health^24^. In this scenario, more comprehensive screening programs could be an alternative, considering that they are currently restricted to blood donors^25^ or restricted in geographical scope^26^. Although there is no treatment for HTLV-1/-2 infection and progression to active disease is rare, a focus on pregnant women could be helpful in reducing vertical transmission of the virus. It has been demanded of the Brazilian government to include HTLV screening in the standard range of tests offered during pre-natal care in the public health system. Therefore, obtaining a national estimate of HTLV-1/-2 prevalence in pregnant women is needed to assess the possible benefits of implementing such a wide screening program. An estimate of prevalence generated through meta-analysis could be an adequate way to approximate national prevalence figures. As such, this systematic review aimed to estimate the prevalence of HTLV-1/-2 infection in pregnant women in Brazil.

## Methods and analysis

### Protocol and registration

This systematic review was prepared in accordance with the Preferred Reporting Items for Systematic Reviews and Meta-Analysis (PRISMA) statement^27^. The protocol for this review has been registered in the PROSPERO International Prospective Register of Systematic Reviews under registration number CRD42019147362 (https://www.crd.york.ac.uk/prospero/display_record.php?ID=CRD42019147362).

### Literature search

We conducted searches in the following databases: the Cochrane Library/CENTRAL, PubMed/MEDLINE, EMBASE, Scopus, BVS/LILACS and Web of Knowledge (ISI). The search was not restricted by date, language or publication type. Search terms included relevant terms in the title, abstract and text, such as Human T-lymphotropic Virus, Pregnant Women and Brazil. The search strategy is presented in online Supplementary File S1.

We searched the reference lists of the studies included in our review to identify additional publications. Additionally, we searched gray literature employing multiple strategies. We searched the website “bancodeteses.capes.gov.br”, a repository of Brazilian theses and dissertations, and the “Open Grey” (http://www.opengrey.eu/). Furthermore, we contacted the technical staff of the Department of Chronic Conditions and Sexually Transmitted Infections of the Brazilian Ministry of Health and experts in the area to gather information from ongoing or unpublished studies. Although this strategy was adopted, no study of this nature (ongoing or unpublished) was found and used in the systematic review. Data from conference proceedings were included. Authors were contacted wherever necessary to assess inconsistent or missing data on papers.

### Eligibility criteria

This systematic review of the literature included studies that met the following criteria: (1) cohort studies or cross-sectional studies; (2) studies assessing HTLV infection in pregnant women in Brazil; and (3) studies with confirmatory tests for HTLV-1/-2. No age, language or date restrictions were applied.

### Study selection and data extraction

Two reviewers (WJD and BAV) assessed the titles and abstracts of all the studies identified in our search. They were blinded to author and journal names. These researchers then independently performed full-text reviews and data extraction using standardized forms on those studies that fulfilled the eligibility criteria. Discrepancies between the reviewers were resolved by consensus; whenever needed, the opinion of a third reviewer (EMW) was sought. Records were managed with a reference management software.

The following data were extracted from the full text documents: title, authors, publication year, study design, number of participants, characteristics of the population (such as age, race, educational level, geographical region of Brazil), number of women infected with HTLV-1 and HTLV-2, number of women not infected with HTLV, HTLV testing method, HTLV risk factors and comorbidities (such as HIV or syphilis coinfection), time of data collection (pre- or postpartum) and study setting. In cases of duplicate reporting, the study that provided more information was included.

### Risk of bias assessment

The quality of the studies included in this review was assessed with an adapted version of the NIH “Quality Assessment Tool for Observational Cohort and Cross-sectional Studies”. The overall strength of the body of evidence was assessed using the “Grading of Recommendations Assessment, Development and Evaluation” (GRADE) tool, using the principles and domains applied in the quality assessment for prognostic studies^28^.

### Statistical analysis

The narrative synthesis of the studies included in this review was structured around the prevalence of HTLV-1/-2 in pregnant women. We considered for meta-analysis only studies with confirmatory tests with typing for HTLV-1/-2. We used a random effects model where the studies weights were assigned by the inverse of their variance (inverse variance method) and the summary measure was obtained by logit transformation. This model was used to pool prevalence data and estimate the prevalence with 95% confidence intervals (CIs). In addition, prediction interval was presented.

We performed a subgroup analysis by geographical region (North, Northeast, Center-West, Southeast and South) into account. Heterogeneity was assessed by means of the chi-square test and the I^2^ statistic. Meta-regression was performed to investigate the effect of age on the prevalence estimates. Cumulative meta-analysis was conducted to assess the influence of time on the prevalence of HTLV-1/-2. All analyses were performed using R 3.6.1 and the packages meta 4.9-7^29^ and metafor 2.1-0^30^.

## Results

**Figure 1:**
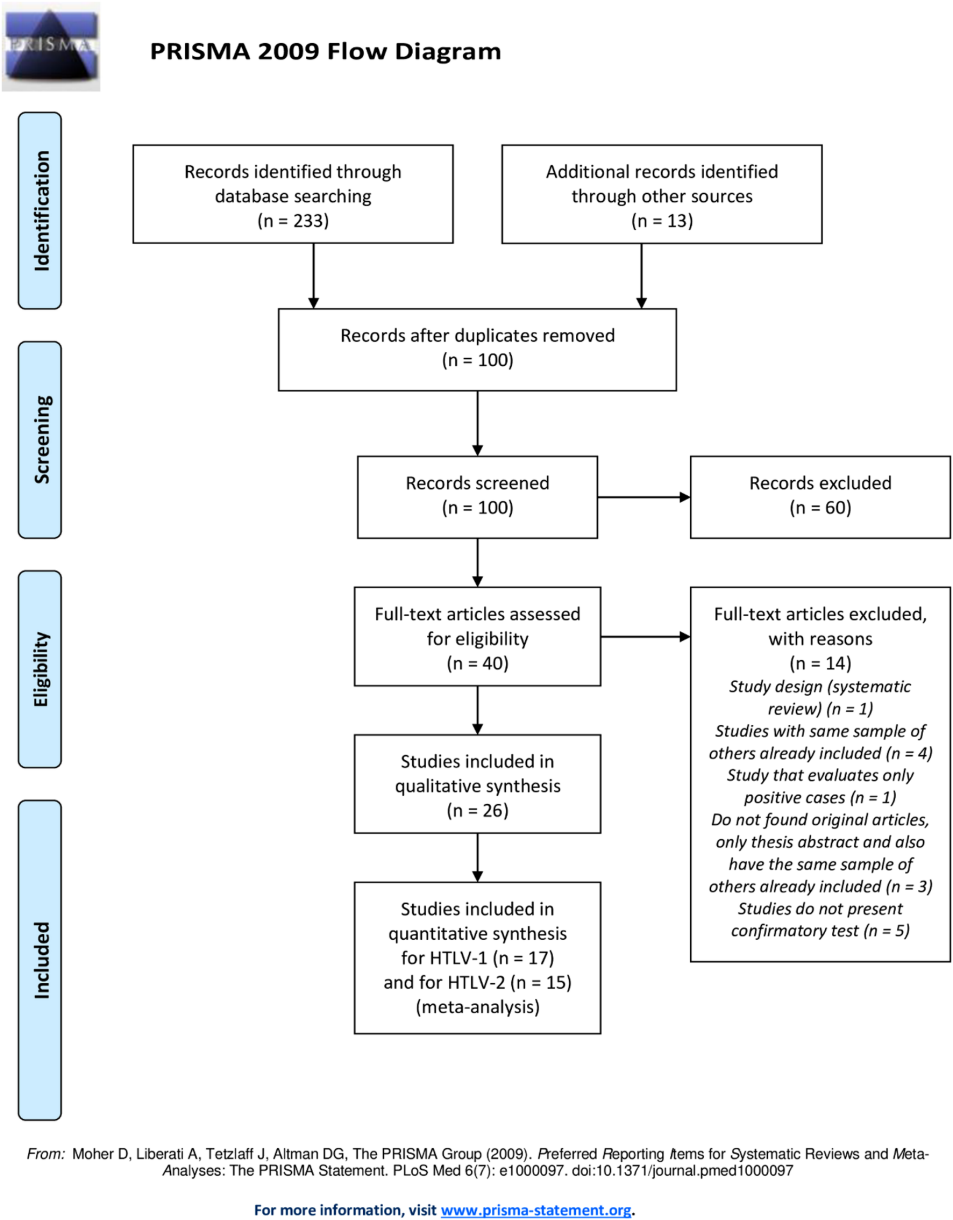
PRISMA flowchart.

We identified 233 abstracts from the selected databases, and 13 additional studies were included from the listed references of the identified manuscripts as well as other sources. From the 100 unique citations, 60 were excluded, and the full texts of the remaining 40 publications were screened. We excluded 14 articles in the subsequent full-text assessments for reasons such as same populations of articles already included, incomplete data, study designs not included in the inclusion criteria and studies do not present confirmatory test (Fig. 1). After this evaluation, 26 articles reporting the prevalence of HTLV were retained for qualitative synthesis^21,26,31–54^. Of these 26 studies evaluated that used confirmatory tests, only 17 reported which types of HTLV were studied and used for quantitative analysis (meta-analysis). Fifteen articles presents data for HTLV-1 and -2^21,26,33,35,39,40,42,45,46,48–52,54^ and two about HTLV-1^43,44^. None report data just for HTLV-2.

A summary of the studies characteristics is presented in Supplementary Table S1. The studies evaluated the prevalence of HTLV in all five regions of Brazil. Regarding screening and confirmatory methods without HTLV typing differentiation, 84.6% of the studies used ELISA, and 80.8% used Western Blot. Of these studies, ten used ELISA and Western-Blot, three of them used ELISA and PCR and eight used ELISA plus Western-Blot and PCR combined.

The overall quality of evidence for HTLV prevalence was rated according to the NIH “Quality Assessment Tool for Observational Cohort and Cross-sectional Studies”, as shown in Supplementary Table S2. In general, the quality of evidence is good. In the point “Was a sample size justification, power description, or variance and effect estimates provided?” the majority the answers are no, but that is due to the fact that the studies are mostly observational, not aimed to find associations, having a more exploratory character. In the point “Was the participation rate of eligible persons at least 50%?” and “Were the outcome assessors blinded to the exposure status of participants?” most studies did not report any explanation.

**Figure 2:**
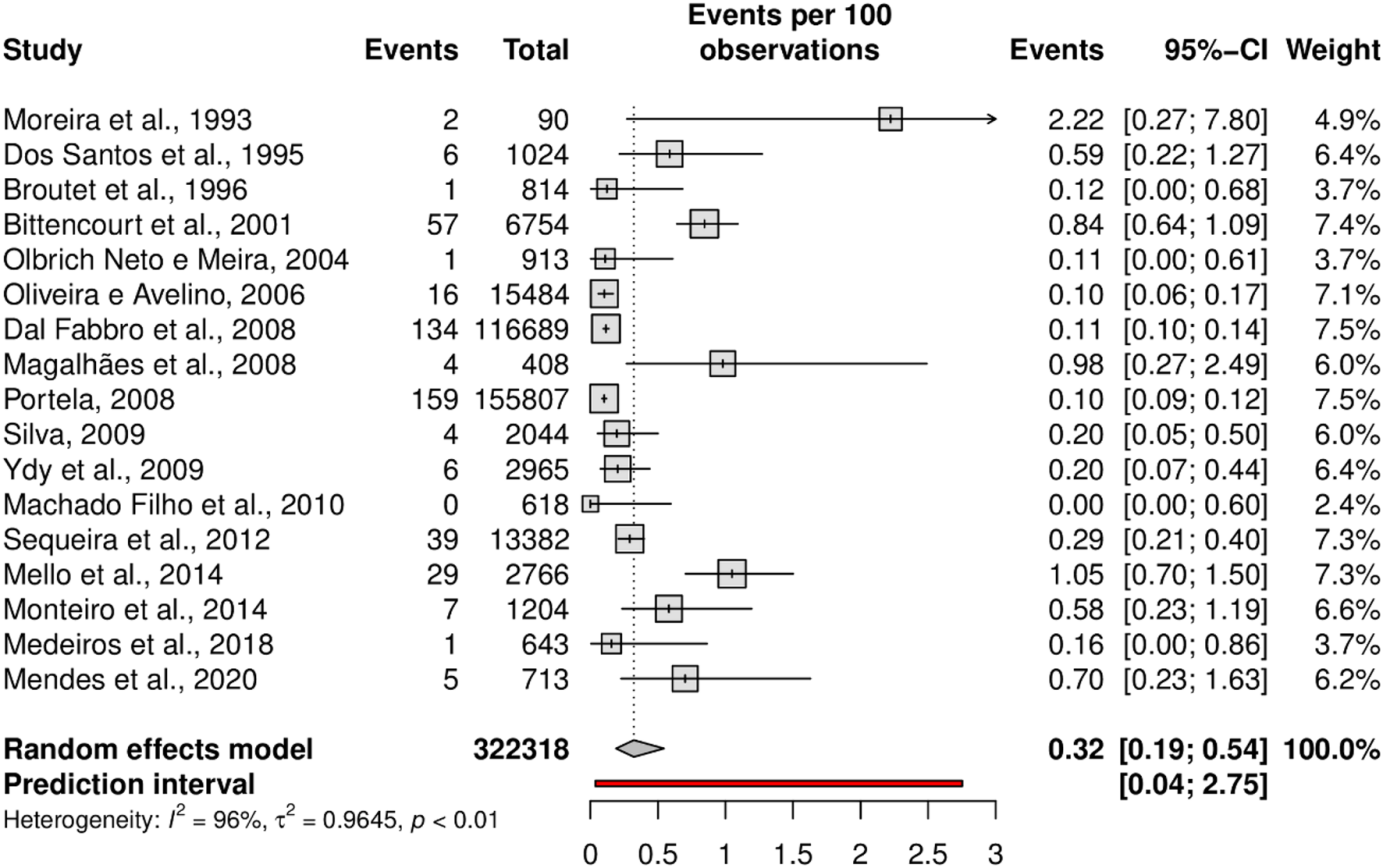
Forest plot of the prevalence of HTLV-1 infection, as detected by confirmatory tests, in pregnant women.

**Figure 3:**
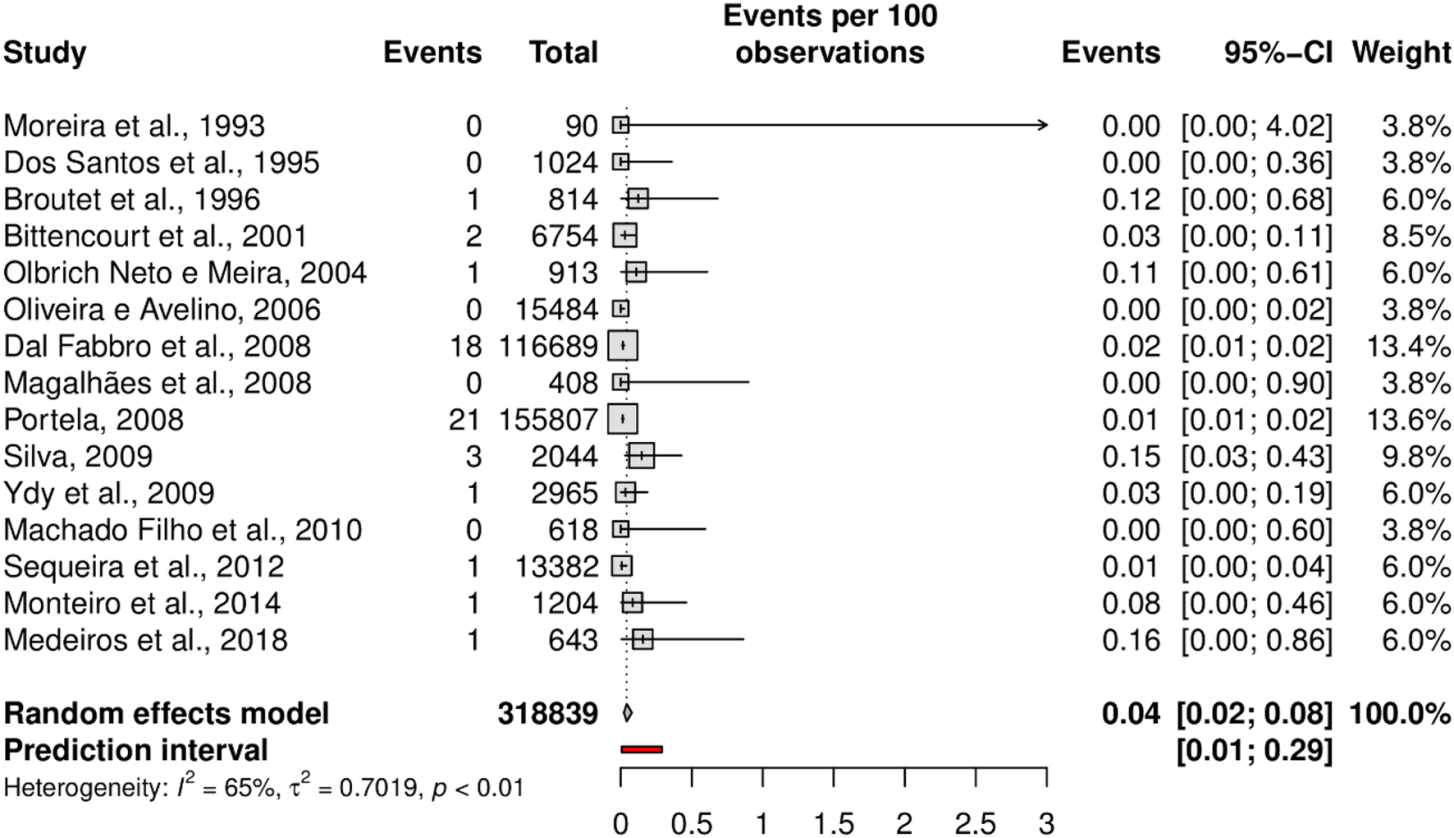
Forest plot of the prevalence of HTLV-2 infection, as detected by confirmatory tests, in pregnant women.

The overall prevalence of HTLV-1 was 0.32% (95% CI 0.19–0.54; 17 studies; I^2^ = 96%). The prediction interval for HTLV-1 prevalence ranged from 0.04 to 2.75%, with 95% confidence (Fig. 2). In addition, the overall prevalence of HTLV-2 was 0.04% (95% CI 0.02–0.08; 15 studies; I^2^ = 65%). The prediction interval for HTLV-2 prevalence ranged from 0.01 to 0.29%, with 95% confidence (Fig. 3). The prediction intervals represent the range of expected HTLV-1 and 2 prevalence in brazilian pregnant women in 95% of settings.

The cumulative meta-analysis showed no important changes in prevalence of HTLV-1 and -2 since 2006 (k = 6) and 2008 (k = 7), respectively (see Supplementary Figs. S1 and S2). It is worth noting that confirmatory PCR tests started to be used in 2001 together with Western-blot or in isolation, according to our subgroup analysis by test type (see Supplementary Figs. S3 and S4). The introduction of PCR as a method to analyze HTLV-1/-2 might lead to higher prevalence estimates, due to an increase in sensitivity^55–57^. However, as most protocols are established in-house and will not have an extensive validation^22^, this can also be a source of heterogeneity between estimated prevalence standard PCR assays for HTLV-1/-2^58^.

**Figure 4:**
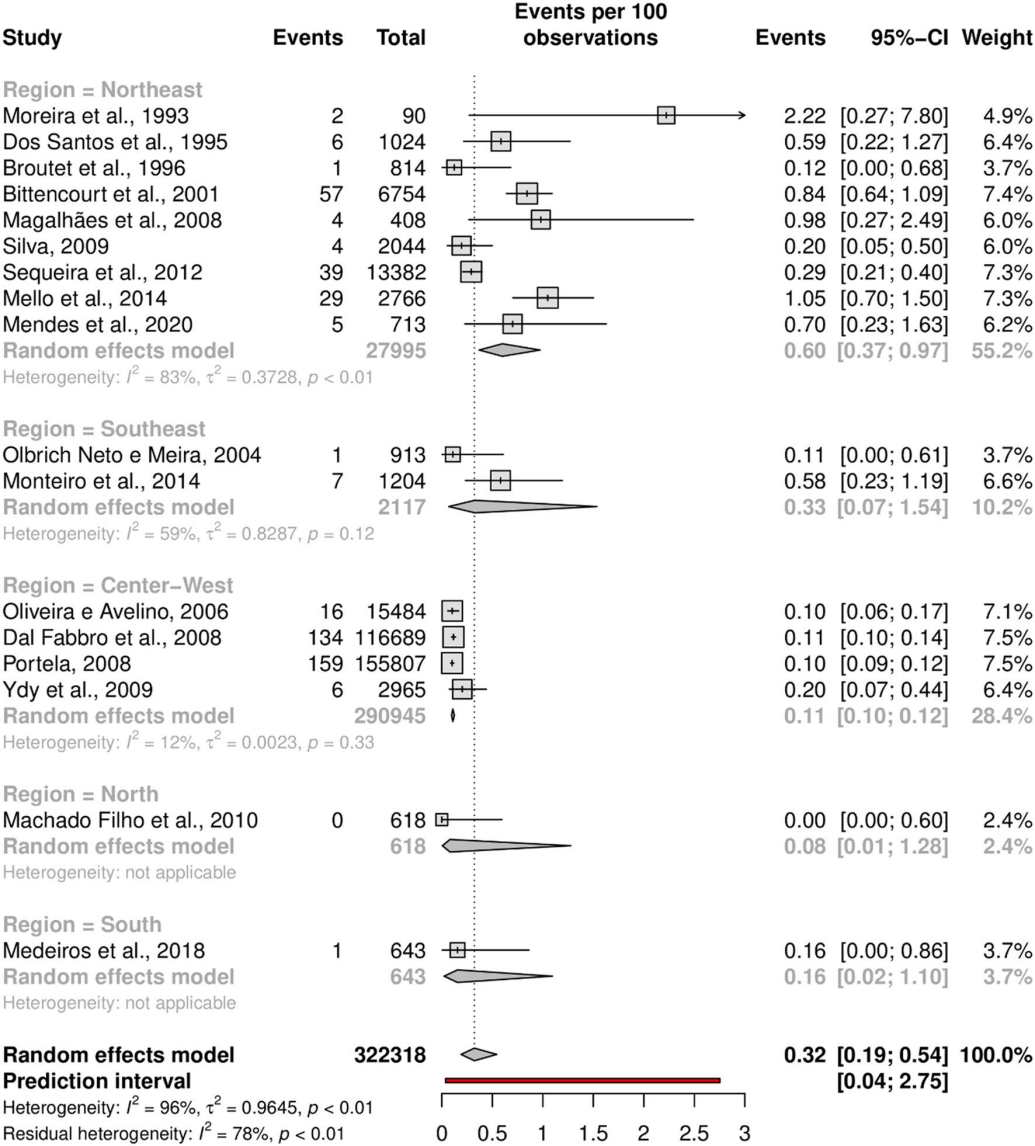
Forest plot of HTLV-1 infection in pregnant women by Brazilian geographical region.

**Figure 5:**
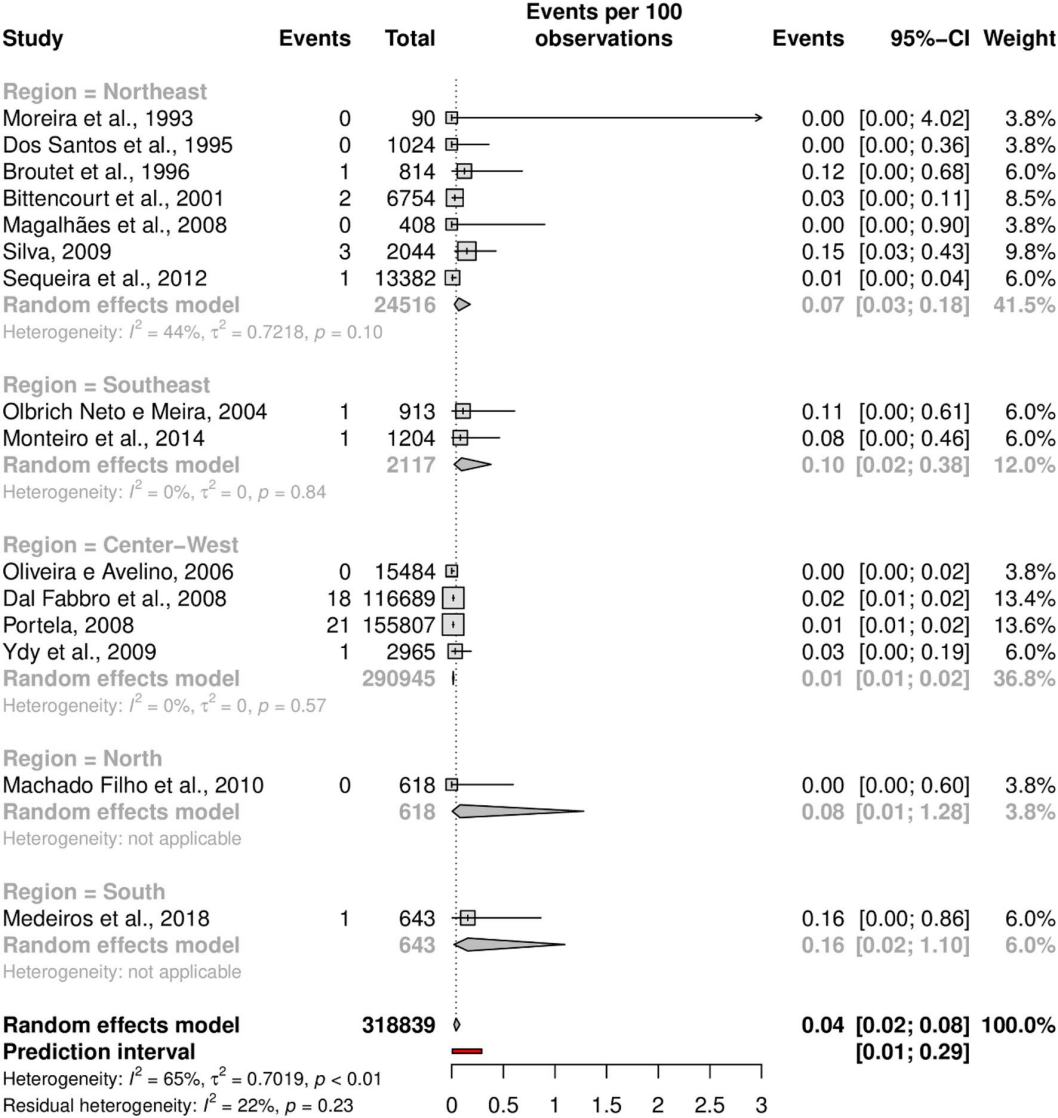
Forest plot of HTLV-2 infection in pregnant women by Brazilian geographical region.

Subgroup analysis was performed in an attempt to explain the heterogeneity among the studies. Looking data for HTLV-1, most studies were carried out in the Northeast region (n = 9), followed by the Center-West (n = 4) and Southeast (n = 2) regions; the North and South regions were represented by one study in each region. Studies for HTLV-2 have the same distribution, unless the Northeast region (n = 7). The forest plots with the estimates for each region are shown in Figs. 4 and 5. The results show generally narrow CIs and a significantly lower prevalence of HTLV-1 and -2 in the Center-West region than in the Northeast region. The point estimate of HTLV-1 prevalence was higher in the Northeast, while HTLV-2 was higher in the South region.

The heterogeneity was high in the Northeast region (I^2^ = 83%), moderate in the Southeast (I^2^ = 59%) and low in the Center-West (I^2^ = 12%), for HTLV-1 and moderate for HTLV-2, in Northeast (I^2^ = 44%). There was no heterogeneity in other regions. It was not possible to do subgroup analysis for North and South regions because there was just one study in each region, with very limited sample size, 618 and 643, respectively and the southern study evaluated high-risk pregnant women. Thus, the analysis results for the North and South should be interpreted with caution. The Northeast region heterogeneity was the only statistically significant (*p* < 0.01) for HTLV-1.

Additional subgroup analyses are presented in the supplemental figures. A comparison of studies that utilized different test methods is presented in Supplementary Figures S3 and S4. The prevalence estimates were not significantly different, but the heterogeneity was lower in studies that used only PCR than in studies that used other methods, for those which evaluated HTLV-1 (Supplementary Fig. S3). Heterogeneity was lower in studies that used only Western-blot for HTLV-2 studies (Supplementary Fig. S4). The versions of the WB assay have evolved over time and more recently PCR started to be more used. The type of confirmatory test used appears to influence different ways for each type of HTLV.

Furthermore, a meta-regression was performed with age as a covariate if the studies described this data. Few studies reported the mean age of their sample (5 of them of HTLV-1 and 4 of them of HTLV-2). This model showed no statistically significant association between HTLV infection and age in pregnant Brazilian women (*p* = 0.217 for HTLV-1 and *p* = 0.131 for HTLV-2).

## Discussion

Overall, we found that the prevalence of HTLV-1 in pregnant women in Brazil is much higher than HTLV-2 with important differences between regions. The prevalence of both HTLV-1 and -2 are higher in the Northeast compared to Center-West region. Studies included in the systematic review were, in general, small and restricted to specific cities or regions and presented a high level of heterogeneity.

Brazil does not have a nationwide screening program for HTLV-1/-2. The state of Mato Grosso do Sul has a comprehensive statewide screening program for sexually transmitted infections in pregnant women, with a low prevalence of HTLV-1/-2 infection^26,50,59^. In 2012, the state of Bahia implemented the “State Prenatal Screening Program on filter paper”, to detect various diseases, including HTLV-1/-2, during the gestational period^60,61^. Selected studies on the prevalence of HTLV in Brazil show a prevalence of over 1% for HTLV-1, particularly in the Northeast region or in specific populations. We found that most studies reporting a high prevalence had small or very localized samples^34,40,43,46^. Conversely, findings of particularly low rates of HTLV-1/-2 infection were common in large studies that analyzed data from the Mato Grosso do Sul screening program^26,50,59^. Differences in the prevalence from the Mato Grosso screening program and other regional studies can result from differences in population included in those studies. Differences in estimated prevalence could be due to real differences about included population or selection bias of some studies, where high risk population is included only.

Due to the highly localized, endemic nature of HTLV infection foci, Brazilian pregnant women must be compared to women from other endemic, high-prevalence countries. Japan is a country that presents one of the areas with the highest prevalence of HTLV-1 in the world^6,62^. Several prevalence studies have used confirmatory tests. In the past 10 years, the Ministry of Health, Labor, and Welfare in Japan has included HTLV-1 antibody testing in antenatal pregnancy screening^63,64^. In 2013, the positivity rates of HTLV-1 Western Blot tests ranged between 0.067% in Kanto (around Tokyo) to 0.663% in Kyushu (southwest area)^65^. The prevalence of this infection in pregnant Japanese women seems to be similar to that in pregnant women in Brazil. However, it seems our estimates are lower than those in pregnant women in other locations. In general, studies from Africa^66–73^ reported prevalence higher than our estimate, ranging around 2.0%, with the highest prevalence of HTLV-1, at 5.5%, in pregnant women who attended an antenatal clinic in Nigeria^72^. Our estimates of HTLV-1 were also lower than those in pregnant women who emigrated from endemic areas, such as the Caribbean (1.42%)^74^. In other endemic countries in Latin America, studies showed prevalence rates of HTLV-1 close to 2.0% in Peru and Jamaica^75–77^ and approximately 3.4% in French Guiana (with significant variations between ethnic groups)^78^.

Among pregnant women who live in Spain, prevalence estimates of HTLV-2 infection range from 0.01 to 0.03%^79,80^. African studies show prevalence of this virus rates between 0.08 and 3.8% in pregnant women^66,68,72,73^. An Argentine study show a prevalence of 0.12% in this population^81^. Although our HTLV-2 estimates seem to be higher than native and immigrant pregnant women who live in Spain, we found lower estimates than other countries.

Of all studies included in this systematic review, only 5 of HTLV-1 and 4 of HTLV-2 reported the mean age of the participants^26,39,44,52,54^, and the range of mean ages was very small (23.9 to 27.0). According to the meta-regression model, age was not associated with the prevalence of HTLV infection, in contrast with the literature. The prevalence increases gradually with age^6,71,82,83^ and is significantly higher in pregnant women older than 30^67^ and 40 years old^70^. Over the years, sexual activity among women of childbearing age increases, suggesting male-to-female transmission^67^.

This study has some limitations that must be acknowledged. While we tried to explore and account for the differences between regions in Brazil, it is very likely that our overall estimates are not representative of the pregnant population across all of Brazil. The included studies did not cover all the states in the country, offering limited estimates of HTLV-1/-2 for each region. In addition to real differences in the prevalence of infection, regions also differed in the number of studies, type of population included in these studies and typical sample size of studies, leading to high heterogeneity due to studies intrinsic factors. Additionally, few studies were population based.

Compared to other infections among pregnant women, HTLV-1 appears to have a similar prevalence to HIV (0.38%)^84^ and hepatitis B (0.4%)^85^. While there is an established national surveillance program for the last two, there is no national screening for HTLV-1. The similarity in prevalence could reflect either true differences in estimates or lack of nationwide information. Since 1993 serologic tests for HTLV-1/-2 became mandatory in Brazilian blood banks^86^, with prevalence ranging between 0.03 and 0.9^87,88^. Those data could suggest a meaning to start a screening program during pregnancy, as was done in Japan. Our study highlights a lack of data on prevalence in pregnant women and the need for nationally and regionally representative measures of burden of HTLV-1/-2 infection in Brazil to support decision-making about screening to prevent vertical transmission.

## Supplementary Information

Supplementary Information 1.

## References

[1] Poiesz BJ, Ruscetti FW, Gazdar AF (1980). Detection and isolation of type C retrovirus particles from fresh and cultured lymphocytes of a patient with cutaneous T-cell lymphoma. Proc. Natl. Acad. Sci. U. S. A..

[2] Kalyanaraman V, Sarngadharan M, Robert-Guroff M (1982). A new subtype of human T-cell leukemia virus (HTLV-II) associated with a T-cell variant of hairy cell leukemia. Science.

[3] Calattini S, Chevalier S, Duprez R (2005). Discovery of a new human T-cell lymphotropic virus (HTLV-3) in Central Africa. Retrovirology.

[4] Wolfe ND, Heneine W, Carr JK (2005). Emergence of unique primate T-lymphotropic viruses among central African bushmeat hunters. Proc. Natl. Acad. Sci..

[5] Verdonck K, González E, Van Dooren S (2007). Human T-lymphotropic virus 1: Recent knowledge about an ancient infection. Lancet Infect. Dis..

[6] Gessain A, Cassar O (2012). Epidemiological aspects and world distribution of HTLV-1 infection. Front. Microbiol..

[7] Koga Y, Iwanaga M, Soda M (2010). Trends in HTLV-1 prevalence and incidence of adult T-cell leukemia/lymphoma in Nagasaki, Japan. J. Med. Virol..

[8] Iwanaga M (2020). Epidemiology of HTLV-1 infection and ATL in Japan: An update. Front. Microbiol..

[9] Treviño A, Aguilera A, Caballero E (2012). Trends in the prevalence and distribution of HTLV-1 and HTLV-2 infections in Spain. Virol. J..

[10] Gonçalves DU, Proietti FA, Ribas JGR (2010). Epidemiology, treatment, and prevention of human T-cell leukemia virus type 1-associated diseases. Clin. Microbiol. Rev..

[11] Proietti FA, Carneiro-Proietti ABF, Catalan-Soares BC (2005). Global epidemiology of HTLV-I infection and associated diseases. Oncogene.

[12] Schierhout G, McGregor S, Gessain A (2020). Association between HTLV-1 infection and adverse health outcomes: A systematic review and meta-analysis of epidemiological studies. Lancet Infect. Dis..

[13] Ishak R, Machado LFA, Cayres-Vallinoto I (2017). Infectious agents as markers of human migration toward the Amazon region of Brazil. Front. Microbiol..

[14] Roucoux DF, Murphy EL (2004). The epidemiology and disease outcomes of human T-lymphotropic vitus type II. AIDS Rev..

[15] Perzova R, Benz P, Abbott L (2010). No evidence of HTLV-3 and HTLV-4 infection in New York state subjects at risk for retroviral infection. AIDS Res. Hum. Retrovir..

[16] Duong YT, Jia H, Lust JA (2008). Absence of evidence of HTLV-3 and HTLV-4 in patients with large granular lymphocyte (LGL) leukemia. AIDS Res. Hum. Retrovir..

[17] Murphy EL, Hanchard B, Figueroa JP (1989). Modelling the risk of adult T-cell leukemia/lymphoma in persons infected with human T-lymphotropic virus type I. Int. J. Cancer.

[18] Nunes D, Boa-Sorte N, Grassi MFR (2017). HTLV-1 is predominantly sexually transmitted in Salvador, the city with the highest HTLV-1 prevalence in Brazil. PLoS ONE.

[19] Tsuji Y, Doi H, Yamabe T (1990). Prevention of mother-to-child transmission of human T-lymphotropic virus type-I. Pediatrics.

[20] Hino S (2011). Establishment of the milk-borne transmission as a key factor for the peculiar endemicity of human T-lymphotropic virus type 1 (HTLV-1): The ATL prevention program Nagasaki. Proc. Jpn. Acad., Ser. B.

[21] Bittencourt AL, Dourado I, Bastos Filho P (2001). Human T-cell lymphotropic virus type 1 infection among pregnant women in northeastern Brazil. J. Acquir. Immun. Defic. Syndr..

[22] Rosadas C, Brites C, Arakaki-Sanchez D (2021). Protocolo Brasileiro para Infecções Sexualmente Transmissíveis 2020: infecção pelo vírus linfotrópico de células T humanas (HTLV). Rev. Soc. Bras. Med. Trop..

[23] Brasil. Ministério da Saúde. Secretaria de Atenção à Saúde. Departamento de Atenção Básica. *Cadernos de Atenção Básica: Atenção ao pré-natal de baixo risco*. (Editora do Ministério da Saúde, 2012).

[24] Brasil. Ministério da Saúde. Portaria de Consolidação N° 4, de 28 de setembro de 2017: Consolidação das normas sobre os sistemas e os subsistemas do Sistema Único de Saúde (2017).

[25] Brasil. Ministério da Saúde. Portaria N° 158, de 04 de fevereiro de 2016: Redefine o regulamento técnico de procedimentos hemoterápicos (2016).

[26] Dal Fabbro MMFJ, da Cunha RV, Bóia MN (2008). Infecção pelo HTLV 1/2: atuação no pré-natal como estratégia de controle da doença no Estado de Mato Grosso do Sul. Rev. Soc. Bras. Med. Trop..

[27] Moher D, Shamseer L, PRISMA-P Group (2015). Preferred reporting items for systematic review and meta-analysis protocols (PRISMA-P) 2015 statement. Syst. Rev..

[28] Iorio A, Spencer FA, Falavigna M (2015). Use of GRADE for assessment of evidence about prognosis: Rating confidence in estimates of event rates in broad categories of patients. BMJ.

[29] Schwarzer G (2007). meta: An R package for meta-analysis. R News.

[30] Viechtbauer W (2010). Conducting meta-analysis in R with the metafor package. J. Stat. Softw..

[31] Andrade CA, Lima Martins MV, Costa JO (1999). Soroprevalência do HIV-1/2, HTLV-I/II e hepatites B e C em parturientes da Maternidade Odete Valadares, Belo Horizonte, Minas Gerais. Rev. Patol. Trop..

[32] Barmpas DBS, Monteiro DLM, Taquette SR (2019). Pregnancy outcomes and mother-to-child transmission rate in HTLV-1/2 infected women attending two public hospitals in the metropolitan area of Rio de Janeiro. PLoS Negl. Trop. Dis..

[33] Broutet N, de Queiroz SA, Basilio FP (1996). Prevalence of HIV-1, HIV-2 and HTLV antibody, in Fortaleza, Ceara, Brazil, 1993–1994. Int. J. STD AIDS.

[34] Costa GB, De Oliveira MC, Gadelha SR (2018). Infectious diseases during pregnancy in Brazil: Seroprevalence and risk factors. J. Infect. Dev. Ctries..

[35] dos Santos JI, Lopes MA, Deliège-Vasconcelos E (1995). Seroprevalence of HIV, HTLV-I/II and other perinatally-transmitted pathogens in Salvador, Bahia. Rev. Inst. Med. Trop. Sao Paulo.

[36] Figueiró-Filho EA, Lopes AHA, de Senefonte FRA (2005). Infecção pelo vírus linfotrópico de células T humanas e transmissão vertical em gestantes de estado da Região Centro-Oeste do Brasil. Rev. Bras. Ginecol. Obstet..

[37] Guerra AB, Siravenha LQ, Laurentino RV (2018). Seroprevalence of HIV, HTLV, CMV, HBV and rubella virus infections in pregnant adolescents who received care in the city of Belém, Pará, Northern Brazil. BMC Pregnancy Childbirth.

[38] Loureiro P, Bezerra A, Souza E (1995). HTLV-1 infection in pregnant women in Recife, Northeastern Brazil. J. Acquir. Immun. Defic. Syndr. Hum. Retrovirol..

[39] Machado Filho AC, Sardinha JFJ, Ponte RL (2010). Prevalência de infecção por HIV, HTLV, VHB e de sífilis e clamídia em gestantes numa unidade de saúde terciária na Amazônia ocidental brasileira. Rev. Bras. Ginecol. Obstet..

[40] Magalhães T, Mota-Miranda AC, Alcantara LCJ (2008). Phylogenetic and molecular analysis of HTLV-1 isolates from a medium sized town in Northern of Brazil: Tracing a common origin of the virus from the most endemic city in the country. J. Med. Virol..

[41] Mata EC, Bezerra RM, Júnior AAP (2018). HTLV-1/2 prevalence in two Amazonian communities. J. Virus Erad..

[42] Medeiros ACM, Vidal LRR, Von Linsingen R (2018). Confirmatory molecular method for HTLV-1/2 infection in high-risk pregnant women. J. Med. Virol..

[43] Mello MAG, da Conceição AF, Sousa SMB (2014). HTLV-1 in pregnant women from the Southern Bahia, Brazil: A neglected condition despite the high prevalence. Virol. J..

[44] de Mendes MFC, de Lima JRO, de Melo BO (2020). Molecular detection of human T cell lymphotropic virus type 1 in pregnant women from Maranhão state, Brazil. Braz. J. Microbiol..

[45] Monteiro DLM, Taquette SR, Barmpas DBS (2014). Prevalence of HTLV-1/2 in pregnant women living in the metropolitan area of Rio de Janeiro. PLoS Negl. Trop. Dis..

[46] Moreira EDJ, Ribeiro TT, Swanson P (1993). Seroepidemiology of human T-cell lymphotropic virus type I/II in northeastern Brazil. J. Acquir. Immun. Defic. Syndr..

[47] Moura AA, Mello MJGD, Correia JB (2015). Prevalence of syphilis, human immunodeficiency virus, hepatitis B virus, and human T-lymphotropic virus infections and coinfections during prenatal screening in an urban Northeastern Brazilian population. Int. J. Infect. Dis..

[48] Olbrich Neto J, Meira DA (2004). Soroprevalência de vírus linfotrópico de células T humanas, vírus da imunodeficiência humana, sífilis e toxoplasmose em gestantes de Botucatu - São Paulo - Brasil: fatores de risco para vírus linfotrópico de células T humanas. Rev. Soc. Bras. Med. Trop..

[49] de Oliveira SR, Avelino MM (2006). Soroprevalência do vírus linfotrópico-T humano tipo I entre gestantes em Goiânia, GO, Brasil. Rev. Bras. Ginecol. Obstet..

[50] Portela, P. C. *Prevalência e diagnóstico laboratorial da infecção pelo vírus HTLV em gestantes de Mato Grosso do Sul, no período de 2002 a 2006* (2008).

[51] Sequeira CG, Tamegão-Lopes BP, dos Santos EJM (2012). Descriptive study of HTLV infection in a population of pregnant women from the state of Pará, Northern Brazil. Rev. Soc. Bras. Med. Trop..

[52] Silva CMS, Sousa VG, Pires C (2009). Prevalência de Sorologia Positiva para o HTLV-1 e HTLV-2 em Gestantes Atendidas em Três Serviços Públicos de Pré-Natal, São Luís, Jul/08 a Jul/09. Cad. Pesqui..

[53] Vargas L, Bastos F, Guimarães A (2020). Seroprevalence and factors associated with human immunodeficiency virus, human T lymphotropic virus and hepatitis B/C infections in parturient women of Salvador-Bahia, Brazil. Braz. J. Infect. Dis..

[54] Ydy RRA, Ferreira D, Souto FJD (2009). Prevalência da infecção pelo vírus linfotrópico humano de células T - HTLV-1/2 entre puérperas de Cuiabá, Estado de Mato Grosso, 2006. Rev. Soc. Bras. Med. Trop..

[55] Andrade RG, Ribeiro MA, Namen-Lopes MSS (2010). Evaluation of the use of real-time PCR for human T cell lymphotropic virus 1 and 2 as a confirmatory test in screening for blood donors. Rev. Soc. Bras. Med. Trop..

[56] van der Ryst E, Smith MS, Visagie HMM (1996). Comparison of the polymerase chain reaction and serology for the diagnosis of HTLV-I infection. J. Infect..

[57] Mackay IM, Arden KE, Nitsche A (2002). Real-time PCR in virology. Nucl. Acids Res..

[58] Waters A, Oliveira ALA, Coughlan S (2011). Multiplex real-time PCR for the detection and quantitation of HTLV-1 and HTLV-2 proviral load: Addressing the issue of indeterminate HTLV results. J. Clin. Virol..

[59] Figueiró-Filho EA, de Senefonte FRA, Lopes AHA (2007). Freqüência das infecções pelo HIV-1, rubéola, sífilis, toxoplasmose, citomegalovírus, herpes simples, hepatite B, hepatite C, doença de Chagas e HTLV I/II em gestantes, do Estado de Mato Grosso do Sul. Rev. Soc. Bras. Med. Trop..

[60] Secretaria de Saúde do Estado da Bahia (SEAB). Portaria estadual n^o^ 1.313, de 14 de setembro de 2012. Resolve: Autorizar a abertura do Credenciamento n° 008/2012. Diário Oficial Estado da Bahia. 15 e 16 set (2012).

[61] Secretaria de Saúde do Estado da Bahia (SEAB). *Boletim Epidemiológico HTLV 2019* (accessed 21 May 2021); http://www.saude.ba.gov.br/wp-content/uploads/2020/04/BoletimHTLV_2019_n%C2%BA03.pdf.

[62] Watanabe T (2011). Current status of HTLV-1 infection. Int. J. Hematol..

[63] Nishijima T, Shimada S, Noda H (2019). Towards the elimination of HTLV-1 infection in Japan. Lancet Infect. Dis..

[64] Itabashi K, Miyazawa T, Sekizawa A (2020). A nationwide antenatal human T-cell leukemia virus type-1 antibody screening in Japan. Front. Microbiol..

[65] Suzuki S, Tanaka M, Matsuda H (2015). Prevalence of human T-cell leukemia virus type 1 carrier in Japanese pregnant women in 2013. J. Clin. Med. Res..

[66] Collenberg E, Ouedraogo T, Ganamé J (2006). Seroprevalence of six different viruses among pregnant women and blood donors in rural and urban Burkina Faso: A comparative analysis. J. Med. Virol..

[67] Armah HB, Narter-Olaga EG, Adjei AA (2006). Seroprevalence of human T-cell lymphotropic virus type I among pregnant women in Accra, Ghana. J. Med. Microbiol..

[68] Sören A, Dias F, Mendez PJ (1997). HTLV-I and -II infections in a nationwide survey of pregnant women in Guinea-Bissau, West Africa. J. Acquir. Immun. Defic. Syndr. Hum. Retrovirol..

[69] Zehender G, Ebranati E, Maddalena CD (2008). Description of a “Trans-Saharan” strain of human T-lymphotropic virus type 1 in West Africa. JAIDS J. Acquir. Immun. Defic. Syndr..

[70] Denis F, Verdier M, Chout R (1988). Prévalence du virus HTLV-1 chez les femmes enceintes en Afrique noire, en Martinique, et d’origine étrangère vivant en France. Bulletin de l’Academie Nationale de Medecine.

[71] Verdier M, Denis F, Sangare A (1989). Prevalence of antibody to human T cell leukemia virus type 1 (HTLV-I) in populations of Ivory Coast, West Africa. J. Infect. Dis..

[72] Olaleye DO, Ekweozor CC, Sheng Z (1995). Evidence of serological cross-reactivities with human immunodeficiency virus types 1 and 2 and human T-lymphotropic virus types I and II in Sera of pregnant women in Ibadan, Nigeria. Int. J. Epidemiol..

[73] Etenna SL-D, Caron M, Besson G (2008). New insights into prevalence, genetic diversity, and proviral load of human T-cell leukemia virus types 1 and 2 in pregnant women in Gabon in Equatorial Central Africa. J. Clin. Microbiol..

[74] Ades AE, Parker S, Walker J (2000). Human T cell leukaemia/lymphoma virus infection in pregnant women in the United Kingdom: Population study. BMJ.

[75] Alarcón JO, Friedman HB, Montano SM (2006). High endemicity of human T-cell lymphotropic virus type 1 among pregnant women in Peru. JAIDS J. Acquir. Immun. Defic. Syndr..

[76] Zurita S, Costa C, Watts D (1997). Prevalence of human retroviral infection in Quillabamba and Cuzco, Peru: A new endemic area for human T cell lymphotropic virus type 1. Am. J. Trop. Med. Hyg..

[77] Dowe G, King SD, Smikle MF (1998). Prevalence of viral and bacterial sexually transmitted pathogens in Jamaican pregnant women. West Indian Med. J..

[78] Tortevoye P, Peneau C, Gessain A (2005). Comparative trends of seroprevalence and seroincidence rates of human T cell lymphotropic virus type I and human immunodeficiency virus 1 in pregnant women of various ethnic groups sharing the same environment in French Guiana. Am. J. Trop. Med. Hyg..

[79] Treviño A, Aguilera A, Caballero E (2009). Seroprevalence of HTLV-1/2 infection among native and immigrant pregnant women in Spain. AIDS Res. Hum. Retrovir..

[80] Treviño A, Benito R, Caballero E (2011). HTLV infection among foreign pregnant women living in Spain. J. Clin. Virol..

[81] Berini CA, Delfino C, Torres O (2013). HTLV-1 cosmopolitan and HTLV-2 subtype b among pregnant women of non-endemic areas of Argentina. Sex Transm. Infect..

[82] Cook LBM, Taylor GP (2014). HTLV-1 and HTLV-2 prevalence in the United States. J. Infect. Dis..

[83] Eshima N, Iwata O, Iwata S (2009). Age and gender specific prevalence of HTLV-1. J. Clin. Virol..

[84] Pereira G, Sabidó M, Caruso A (2016). HIV prevalence among pregnant women in Brazil: A national survey. Rev. Bras. Ginecol. Obstet..

[85] Brasil. Ministério da Saúde. Secretaria de Vigilância em Saúde. Boletim Epidemiológico de Hepatites Virais (2020).

[86] Brasil. Ministério da Saúde. Portaria n^o^ 1.376, de 19 de novembro de 1993. Aprova alterações na Portaria n^o^ 721/GM, de 09.08.89, que aprova Normas Técnicas para coleta, processamento e transfusão de sangue, componentes e derivados, e dá outras providências. Diário Oficial da União 02 dez (1993).

[87] Pinto MT, Rodrigues ES, Malta TM (2012). HTLV-1/2 seroprevalence and coinfection rate in Brazilian first-time blood donors: An 11-year follow-up. Rev. Inst. Med. Trop. S. Paulo.

[88] Ishak R, de Ishak MOG, Vallinoto ACR (2020). The challenge of describing the epidemiology of HTLV in the Amazon region of Brazil. Retrovirology.

