# Supplementary material for: Prevalence of human T-lymphotropic virus type 1 and 2 (HTLV-1/-2) infection in pregnant women in Brazil: a systematic review and meta-analysis
Source: Sci Rep. 2021 Jul 28;11:15367. doi: 10.1038/s41598-021-94934-7 (PMC8319321; doi:10.1038/s41598-021-94934-7)
Supplement: Supplementary file 1 — Supplementary Information 1. [file 41598_2021_94934_MOESM1_ESM.pdf]

Prevalence of human T-lymphotropic virus type 1 and 2 (HTLV-1/-2) infection in pregnant women in Brazil: a systematic review and meta-analysis

Bruna Angelo Vieira, Augusto Bacelo Bidinotto, William Jones Dartora, Luana Giongo Pedrotti, Vanessa Martins de Oliveira, Eliana Márcia Wendland*

Supplementary File S1. Details of electronic bibliographic database search strategies

Cochrane Central
(MeSH descriptor: [Pregnant Women] OR "Pregnant Women" OR MeSH descriptor: [Pregnancy] OR Pregnancy OR Pregnant OR MeSH descriptor: [Pregnancy Complications] OR "Pregnancy Complication" OR MeSH descriptor: [Delivery, Obstetric] OR "Delivery, Obstetric" OR MeSH descriptor: [Postpartum Period] OR "Postpartum Period" OR MeSH descriptor: [Prenatal Diagnosis] OR "Prenatal Diagnosis") AND (MeSH descriptor: [Human T-lymphotropic virus 1] OR HTLV-I OR MeSH descriptor: [Human T-lymphotropic virus 2] OR HTLV-II) AND (MeSH descriptor: [Brazil] OR Brazil* OR Brasil*)

Medline (via Pubmed)]
(Human T-lymphotropic virus 1[MeSH Terms]) OR (("Human T-lymphotropic virus") AND ("1" OR "i")) OR ((Human AND ("T-lymphotropic") AND ("virus") AND ("1" OR "i")) OR (("Human T Cell Leukemia Virus") AND ("1" OR "i")) OR ((Human AND ("T Cell") AND (Leukemia) AND (Virus) AND ("1" OR "i")) OR ("HTLV-i") OR ("HTLV-1") OR (("HTLV" AND ("1" OR "i")) OR (Human T-lymphotropic virus 2[MeSH Terms]) OR (("Human T-lymphotropic virus") AND ("2" OR "ii")) OR ((Human AND ("T-lymphotropic") AND ("virus") AND ("2" OR "ii")) OR ((""Human T Cell Leukemia Virus"") AND (""2"" OR ""ii""))) OR ((Human AND ("T Cell") AND (Leukemia) AND (Virus) AND ("2" OR "ii")) OR ("HTLV-ii") OR ("HTLV-2") OR (("HTLV" AND ("2" OR "ii")) OR ("htlv-i antigens"[MeSH Terms]) OR (("htlv"[All Fields] AND ("i"[All Fields] OR "1"[All Fields])) OR (("htlv-i"[All Fields] OR "htlv-1"[All Fields]) AND "antigens"[All Fields])) OR (("htlv-ii antigens"[MeSH Terms]) OR ("htlv"[All Fields] AND ("ii"[All Fields] OR "2"[All Fields]))) OR (("htlv-ii"[All Fields] OR "htlv-2"[All Fields]) AND "antigens"[All Fields]) OR ("htlv-i antibodies"[MeSH Terms] OR ("htlv"[All Fields] AND ("i"[All Fields] OR "1"[All Fields]))) OR (("htlv-i"[All Fields] OR "htlv-1"[All Fields]) AND "antibodies"[All Fields]) OR ("htlv-ii antibodies"[MeSH Terms] OR ("htlv"[All Fields] AND ("ii"[All Fields] OR "2"[All Fields]))) OR (("htlv-ii"[All Fields] OR "htlv-2"[All Fields]) AND "antibodies"[All Fields]) OR ("htlv"[All Fields] AND ("i"[All Fields] OR "1"[All Fields])) OR (("htlv-i"[All Fields] OR "htlv-1"[All Fields]) AND "antibody"[All Fields]) OR ("htlv"[All Fields] AND ("ii"[All Fields] OR "2"[All Fields])) OR (("htlv-ii"[All Fields] OR "htlv-2"[All Fields]) AND "antibody"[All Fields]) OR ("htlv-ii infections"[MeSH Terms] OR (("htlv"[All Fields] AND ("ii"[All Fields] OR "2"[All Fields])) OR (("htlv-ii"[All Fields] OR "htlv-2"[All Fields]) AND "infections"[All Fields])) OR ("htlv-i infections"[MeSH Terms] OR (("htlv"[All Fields] AND ("i"[All Fields] OR "1"[All Fields])) OR (("htlv-i"[All Fields] OR "htlv-1"[All Fields]) AND "infections"[All Fields])) OR deltaretrovirus AND ((pregnant women[MeSH Terms]) OR (pregnancy[MeSH Terms]) OR (pregnancy complication[MeSH Terms]) OR (prenatal diagnosis[MeSH Terms]) OR (postpartum period[MeSH Terms]) OR (delivery, obstetric[MeSH Terms]) OR (delivery) OR (pregnancy) OR (pregnant)) AND (((Brazil*) OR Brasil*) OR Brazil[MeSH Terms]))

BVS/ LILACS
((tw:("pregnant women" OR "pregnancy" OR "pregnant" OR "pregnancy complication" OR "delivery, obstetric" OR "postpartum period" OR "prenatal diagnosis"))
AND
(tw:("HTLV-I" OR "HTLV-II" OR "Human T-lymphotropic Virus-II" OR "Human T-lymphotropic Virus-I" OR "Human T Cell Leukemia Virus-I" OR "Human T Cell Leukemia Virus-II"))
AND
(tw:("Brasil*" OR "Brazil*")))

Web of Knowledge (ISI)
(ALL=(Pregnant Women) OR ALL=(Pregnancy) OR ALL=(Pregnant) OR ALL=(Pregnancy Complications) OR ALL=(Delivery, Obstetric) OR ALL=(Postpartum Period) OR ALL=(Prenatal Diagnosis))
AND
(ALL=(HTLV-I OR HTLV-1 OR Human T-lymphotropic virus I OR Human T-lymphotropic virus 1) OR ALL=(HTLV-II OR HTLV-2 OR Human T-lymphotropic virus II OR Human T-lymphotropic virus 2) OR ALL=(HTLV-I Antigens OR HTLV-II

Antigens OR HTLV-1 Antigens OR HTLV-2 Antigens) OR ALL=(HTLV-I Antibodies OR HTLV-II Antibodies OR HTLV-1 Antibodies OR HTLV-2 Antibodies) OR ALL=(Human T Cell Leukemia Virus-I OR Human T Cell Leukemia Virus-II OR Human T Cell Leukemia Virus-1 OR Human T Cell Leukemia Virus-2))
AND
ALL=(Brasil* OR Brazil*)

EMBASE
('human t-lymphotropic virus 1'/exp OR 'atlv' OR 'adult t cell leukaemia virus' OR 'adult t cell leukemia virus' OR 'htlv 1' OR 'htlv i' OR 'htlv-1' OR 'htlv-i' OR 'htlv1' OR 'human t cell leukaemia virus 1' OR 'human t cell leukemia virus 1' OR 'human t-lymphotropic virus 1' OR 'human t cell leukemia virus i' OR 'human t cell leukemia virus type 1' OR 'human t cell lymphotropic virus type i' OR 'human t lymphotropic virus 1' OR 'human t lymphotropic virus type 1' OR 'human t cell leukaemia virus i' OR 'human t cell leukaemia virus type 1') OR ('human t cell leukemia virus'/exp OR 'htlv' OR 'htlv viruses' OR 'human t cell leukaemia virus' OR 't cell leukaemia virus, human' OR 't cell leukemia virus, human' OR 't lymphocyte leukaemia virus (human)' OR 't lymphocyte leukemia virus (human)' OR 'human t cell leukaemia lymphoma virus' OR 'human t cell leukemia lymphoma virus' OR 'human t cell leukemia virus' OR 'human t lymphocyte leukaemia virus' OR 'human t lymphocyte leukemia virus') OR ('human t cell leukemia virus antigen'/exp OR 'deltaretrovirus antigens' OR 'human t cell leukaemia virus antigen' OR 'human t cell leukemia virus antigen' OR 'htlv antigen' OR 'htlv antigens' OR 'htlv blv antigens' OR 'htlv i antigens' OR 'htlv ii antigens' OR 'htlv-blv antigens' OR 'htlv-i antigens' OR 'htlv-ii antigens') OR ('human t cell leukemia virus antibody'/exp OR 'deltaretrovirus antibodies' OR 'human t cell leukaemia virus antibody' OR 'human t cell leukemia virus antibody' OR 'htlv antibodies' OR 'htlv antibody' OR 'htlv blv antibodies' OR 'htlv i antibodies' OR 'htlv ii antibodies' OR 'htlv-blv antibodies' OR 'htlv-i antibodies' OR 'htlv-ii antibodies') OR ('human t-lymphotropic virus 2'/exp OR 'htlv 2' OR 'htlv ii' OR 'htlv-ii' OR 'htlv2' OR 'human t cell leukaemia virus 2' OR 'human t cell leukemia virus 2' OR 'human t-lymphotropic virus 2' OR 'hairy cell leukaemia virus' OR 'hairy cell leukemia virus')
AND
('pregnancy'/exp OR 'child bearing' OR 'childbearing' OR 'gestation' OR 'gravidity' OR 'intrauterine pregnancy' OR 'labor presentation' OR 'labour presentation' OR 'pregnancy' OR 'pregnancy maintenance' OR 'pregnancy trimesters') OR ('pregnancy complication'/exp OR 'diseases complicating pregnancy' OR 'gestational complication' OR 'pregnancy complication' OR 'pregnancy complications' OR 'pregnancy complications, cardiovascular' OR 'pregnancy complications, haematologic' OR 'pregnancy complications, hematologic' OR 'pregnancy complications, infectious' OR 'pregnancy complications, neoplastic' OR 'pregnancy complications, parasitic') OR ('prenatal diagnosis'/exp OR 'antenatal diagnosis' OR 'diagnosis, antenatal' OR 'diagnosis, prenatal' OR 'prenatal diagnosis')
AND
'brazil'/exp OR 'brazil' OR 'federative republic of brazil' OR brasil

Scopus
(TITLE-ABS-KEY(HTLV-I) OR TITLE-ABS-KEY(HTLV-II) OR TITLE-ABS-KEY(HTLV-1) OR TITLE-ABS-KEY(HTLV-2) OR TITLE-ABS-KEY(HTLV) OR TITLE-ABS-KEY(Human T-Lymphotropic Virus 1) OR TITLE-ABS-KEY(Human T Lymphotropic Virus 1) OR TITLE-ABS-KEY(Human T-Lymphotropic Virus 2) OR TITLE-ABS-KEY(Human T Lymphotropic Virus 2) OR TITLE-ABS-KEY(Human T-Lymphotropic Virus I) OR TITLE-ABS-KEY(Human T Lymphotropic Virus I) OR TITLE-ABS-KEY(Human T-Lymphotropic Virus II) OR TITLE-ABS-KEY(Human T Lymphotropic Virus II) OR TITLE-ABS-KEY(Human T-Cell Leukemia Virus 1) OR TITLE-ABS-KEY(Human T Cell Leukemia Virus 1) OR TITLE-ABS-KEY(Human T-Cell Leukemia Virus 2) OR TITLE-ABS-KEY(Human T Cell Leukemia Virus 2) OR TITLE-ABS-KEY(Human T-Cell Leukemia Virus I) OR TITLE-ABS-KEY(Human T Cell Leukemia Virus I) OR TITLE-ABS-KEY(Human T-Cell Leukemia Virus II) OR TITLE-ABS-KEY(Human T Cell Leukemia Virus II))
AND
TITLE-ABS-KEY(pregnancy) OR TITLE-ABS-KEY(pregnant) OR TITLE-ABS-KEY(postpartum) OR TITLE-ABS-KEY(prenatal) OR TITLE-ABS-KEY(delivery))
AND
TITLE-ABS-KEY(brazil) OR TITLE-ABS-KEY(brazilian) OR TITLE-ABS-KEY(brasil) OR TITLE-ABS-KEY(brasilian))

Supplementary Table S1. Qualitative synthesis of studies reporting the prevalence of HTLV infection in pregnant women in Brazil.

Author, year	Region, State	Population	N	Age (mean)	Age (range)	Educational level	N screening +	Screening method	N confirmatory +	Confirmatory method	HTLV 1	HTLV 2
Andrade et al, 1999 [31]	Southeast, MG	Postpartum women attended at Odete Valadares Maternity from April 1994 to January 1995.	1,959	28.1	-	-	20	ELISA	3	Western Blot	-	-
Barmpas et al, 2019 [32]	Southeast, RJ	Pregnant women enrolled at admission for delivery at two public hospitals in the metropolitan area of Rio de Janeiro, namely, the 'Pedro Ernesto' University Hospital of the Rio de Janeiro State University (Universidade do Estado do Rio de Janeiro–HUPE/UERJ) and the 'Hospital Estadual da Mãe' (HEM), and recruited at their first antenatal visit between November 2012 and December 2017.	1,628	-	Categories<br>≤24<br>>24	Years of schooling:<br>≥10<br><10	14	CMIA—Architect rHTLV-I/II, Abbott	12	Western Blot	-	-
Bittencourt et al, 2001 [21]	Northeast, BA	Pregnant women who attended the prenatal care unit of a state maternity hospital (IPERBA) in the city of Salvador between January 1996 and September 1998.	6,754	-	Categories<br>14-20<br>21-30<br>31-42	Illiterate<br>Literate	61	ELISA	59	Western Blot, PCR	57	2
Broutet et al, 1996 [33]	Northeast, CE	Six selected population groups, including pregnant women recruited from public health centers and public hospitals	814	-	-	Illiterate<br>Did not complete primary school	3	ELISA	2	Western Blot	1	1

Author, year	Region, State	Population	N	Age (mean)	Age (range)	Educational level	N screening +	Screening method	N confirmatory +	Confirmatory method	HTLV 1	HTLV 2
		from July 1993 to February 1994.				Had been to university						
Costa et al, 2018 [34]	Northeast, BA	Pregnant mothers who attended the women reference attention center (Maternidade Santa Helena) in Ilhéus city during July 2009-2010.	511	-	13-44	Have never gone to school<br><br>Elementary school or less<br><br>High school or more	6	ELISA	6	Western Blot, PCR	-	-
Dal Fabbro et al, 2008 [26]	Center-West, MS	Women who sought prenatal care in the Program of Protection to the Pregnant Woman of the State of Mato Grosso do Sul.	116,689	27	14-44	Years of schooling:<br><br>≤3<br><br>4-7<br><br>8-11<br><br>≥12	153	ELISA	153	Western Blot, PCR	134	18
Dos Santos et al, 1995 [35]	Northeast, BA	Pregnant women referred to the Bahia state public health laboratory for prenatal test between November 1990 and June 1991.	1,024	-	Categories<br><21<br>21-30<br>>30	Illiterate<br><br>1-8 school years<br><br>>8 school years	14	ELISA	9	Western Blot	6	0
Figueiró-Filho et al, 2005 [36]	Center-West, MS	Pregnant women screened by the Pregnant Protection Program of the State of Mato Grosso do Sul.	32,512	-	Categories<br>15-19<br>20-29<br>30-39<br>40-49	-	37	ELISA	29	Western Blot, PCR	-	-

Author, year	Region, State	Population	N	Age (mean)	Age (range)	Educational level	N screening +	Screening method	N confirmatory +	Confirmatory method	HTLV 1	HTLV 2
Guerra et al, 2018 [37]	North, PA	Pregnant adolescents who underwent prenatal care at the Reference Unit Specialized in Maternal-Child and Adolescent Care (Unidade de Referência Especializada Materno-Infantil Adolescência - UREMIA) from November 2009 to February 2010.	324	15.8	12-18	Years of education: <6 6-9 >9	-	ELISA	2	PCR	-	-
Loureiro et al, 1995 [38]	Northeast, PE	Pregnant women.	1,000	-	14-40	-	16	ELISA	5	Western Blot	-	-
Machado Filho et al, 2010 [39]	North, AM	Pregnant women of the spontaneous demand of the Amazonian Tropical Medicine Foundation (FMTAM) submitted to prenatal screening from March to September 2008.	618	23.9	13-43	Schooling (years): Illiterate 1-4 5-8 9-11 >11	0	ELISA	0	PCR	0	0
Magalhães et al, 2008 [40]	Northeast, BA	Pregnant women over 10 weeks pregnant from Cruz das Almas were selected randomly between June and October 2005.	408	-	14-32	Years of schooling	4	ELISA	4	Western Blot	4	0

Author, year	Region, State	Population	N	Age (mean)	Age (range)	Educational level	N screening +	Screening method	N confirmatory +	Confirmatory method	HTLV 1	HTLV 2
Mata et al, 2018 [41]	North, AM	Women of reproductive age in the Oiapoque urban area.	216	-	Categories<br>0-15<br>16-20<br>21-30<br>31-40<br>41-50	Basic education<br>Median education<br>Higher education	0	ELISA	0	Western Blot	-	-
Medeiros et al, 2018 [42]	South, PR	Pregnant women who were followed up in a high-risk prenatal care unit at the Hospital de Clínicas/Universidade Federal do Paraná Brazil (HC-UFPR) from August 2015 to August 2016.	643	-	18-47	Years of schooling	4	CMIA (ARCHITECT rHTLV-I/II, Abbott Laboratories, Germany)	2	PCR	1	1
Mello et al, 2014 [43]	Northeast, BA	Women treated at the antenatal units of the two of the largest regional hospitals–one located in Ilheus and the other in Itabuna between November 2008 and May 2010.	2,766	-	Categories<br>9-19<br>20-29<br>>30	Illiterate<br>Literate	34	ELISA	29	Western Blot, PCR	29	-
Mendes et al, 2020 [44]	Northeast, MA	Pregnant volunteers who were selected by free choice during the prenatal period at the LACEN-MA between February 2015 and May 2017.	713	24.3	15-43	< Full high school<br>≥ Full high school	5	CMIA	5	Western Blot, PCR	5	-
Monteiro et al, 2014 [45]	Southeast, RJ	Pregnant women recruited upon admission for delivery in two public hospitals in the metropolitan area of Rio de	1,204	-	Categories<br><24<br>≥25	Years of schooling	10	CMIA—Architect rHTLV-I/II, Abbott	8	Western Blot	7	1

Author, year	Region, State	Population	N	Age (mean)	Age (range)	Educational level	N screening +	Screening method	N confirmatory +	Confirmatory method	HTLV 1	HTLV 2
		Janeiro, between November 2012 and April 2013.										
Moreira et al, 1993 [46]	Northeast, BA	Pregnant women of an obstetric hospital of the state (Instituto de Perinatologia da Bahia) between April 1990 and March 1991.	90	24 (median)	17-44	-	2	ELISA	2	Dot-blot	2	0
Moura et al, 2015 [47]	Northeast, AL	Pregnant women who sought treatment at the Maceio´ Universal Healthcare System (Sistema Único de Saúde, SUS) during their first prenatal care visit between June 2007 and May 2012.	54,798	23.3	15-35 (confidence interval)	-	129	ELISA	118	Western Blot	-	-
Olbrich Neto e Meira, 2004 [48]	Southeast, SP	Pregnant women attended at Basic Health Units in the municipality of Botucatu.	913	-	-	-	2	ELISA	2	Western Blot	1	1
Oliveira e Avelino, 2006 [49]	Center-West, GO	All pregnant women who attended prenatal care in all units of the Municipal Health Department of Goiânia between September 2003 and December 2004.	15,485	-	<30<br>≥30	Years of schooling:<br>≥9<br><9	19	ELISA	16	PCR	16	0
Portela, 2008 [50]	Center-West, MS	Pregnant women screened for diseases by the State Program for the Protection of Pregnant	155,807	-	-	-	265	ELISA	206	Western Blot, PCR	159	21

Author, year	Region, State	Population	N	Age (mean)	Age (range)	Educational level	N screening +	Screening method	N confirmatory +	Confirmatory method	HTLV 1	HTLV 2
		Women of Mato Grosso do Sul from 2002 to 2006.										
Sequeira et al, 2012 [51]	Northern, PA	Pregnant women interviewed during prenatal care consultations in health care units of 19 municipalities who joined the Mother to Child program from February to November of 2008.	13,382	-	Categories 15-19 20-39 40-49	Elementary school<br>High school<br>Higher education<br>Not informed	43	ELISA (Murex HTLV-I+II, Dartford, England)	41	Western Blot	39	1
Silva, 2009 [52]	Northeast, MA	Women with pregnancies up to 22 weeks from three public services, where their prenatal care took place between February to December 2008.	2,044	25.4	18-45	-	7	ELISA	7	Western Blot, PCR	4	3
Vargas et al, 2020 [53]	Northeast, BA	Parturient women attended at two public maternity hospitals in Salvador, Bahia from April 2016 to June 2017.	2,099	27.3	14-46	Years of schooling:<br><8<br>>8	10	ELISA	9	Western Blot, PCR	-	-
Ydy et al, 2009 [54]	Center-West, MT	Postpartum women identified from the hospitalization registry book in three public maternity hospitals or those affiliated with the Cuiabá Unified Health System from April to September 2006.	2,965	23.9	13-44	None<br>Basic education<br>Median education<br>Higher education	9	ELISA	7	Western Blot	6	1

Supplementary Table S2. Quality assessment according to the NIH "Quality Assessment Tool for Observational Cohort and Cross-sectional Studies".

Author, year	Was the research question or objective in this paper clearly stated?	Was the study population clearly specified and defined?	Was the participation rate of eligible persons at least 50%?	Were all the subjects selected or recruited from the same or similar populations (including the same time period)? Were inclusion and exclusion criteria for being in the study prespecified and applied uniformly to all participants?	Was a sample size justification, power description, or variance and effect estimates provided?	Were the outcome measures (dependent variables) clearly defined, valid, reliable, and implemented consistently across all study participants?	Were the outcome assessors blinded to the exposure status of participants?
Andrade et al, 1999 [31]	yes	yes	NR	yes	no	yes	NR
Barmpas et al, 2019 [32]	yes	yes	NR	yes	no	yes	NR
Bittencourt et al, 2001 [21]	yes	yes	yes	yes	no	yes	NR
Broutet et al, 1996 [33]	yes	yes	yes	yes	yes	yes	NR
Costa et al, 2018 [34]	yes	no	NR	yes	no	no	NR
Dal Fabbro et al, 2008 [26]	yes	yes	NR	yes	no	yes	NR
Dos Santos et al, 1995 [35]	yes	yes	NR	yes	yes	yes	NR
Figueiró-Filho et al, 2005 [36]	yes	yes	NR	yes	no	yes	NR
Guerra et al, 2018 [37]	yes	yes	yes	yes	yes	yes	NR
Loureiro et al, 1995 [38]	yes	CD	yes	CD	CD	CD	CD
Machado Filho et al, 2010 [39]	yes	yes	NR	yes	yes	no	NR

Author, year	Was the research question or objective in this paper clearly stated?	Was the study population clearly specified and defined?	Was the participation rate of eligible persons at least 50%?	Were all the subjects selected or recruited from the same or similar populations (including the same time period)? Were inclusion and exclusion criteria for being in the study prespecified and applied uniformly to all participants?	Was a sample size justification, power description, or variance and effect estimates provided?	Were the outcome measures (dependent variables) clearly defined, valid, reliable, and implemented consistently across all study participants?	Were the outcome assessors blinded to the exposure status of participants?
Magalhães et al, 2008 [40]	yes	yes	NR	yes	no	yes	NR
Mata et al, 2018 [41]	yes	yes	NR	no	no	yes	NR
Medeiros et al, 2018 [42]	yes	yes	NR	no	no	yes	NR
Mello et al, 2014 [43]	yes	yes	NR	no	no	yes	NR
Mendes et al, 2020 [44]	yes	yes	NR	yes	no	yes	NR
Monteiro et al, 2014 [45]	yes	yes	NR	yes	yes	yes	NR
Moreira et al, 1993 [46]	yes	yes	NR	yes	no	yes	NR
Moura et al, 2015 [47]	yes	yes	NA	yes	no	yes	NR
Olbrich Neto e Meira, 2004 [48]	yes	yes	NR	yes	yes	yes	NR
Oliveira e Avelino, 2006 [49]	yes	yes	NR	yes	no	yes	NR
Portela, 2008 [50]	yes	yes	yes	yes	no	yes	NR
Sequeira et al, 2012 [51]	yes	yes	NR	yes	no	yes	NR
Silva, 2009 [52]	yes	yes	NR	yes	yes	yes	NR
Vargas et al, 2020 [53]	yes	yes	NR	yes	yes	yes	NR
Ydy et al, 2009 [54]	yes	yes	yes	yes	yes	yes	NR

CD = cannot determine; NA = not applicable; NR = not reported

Supplementary Figure S1

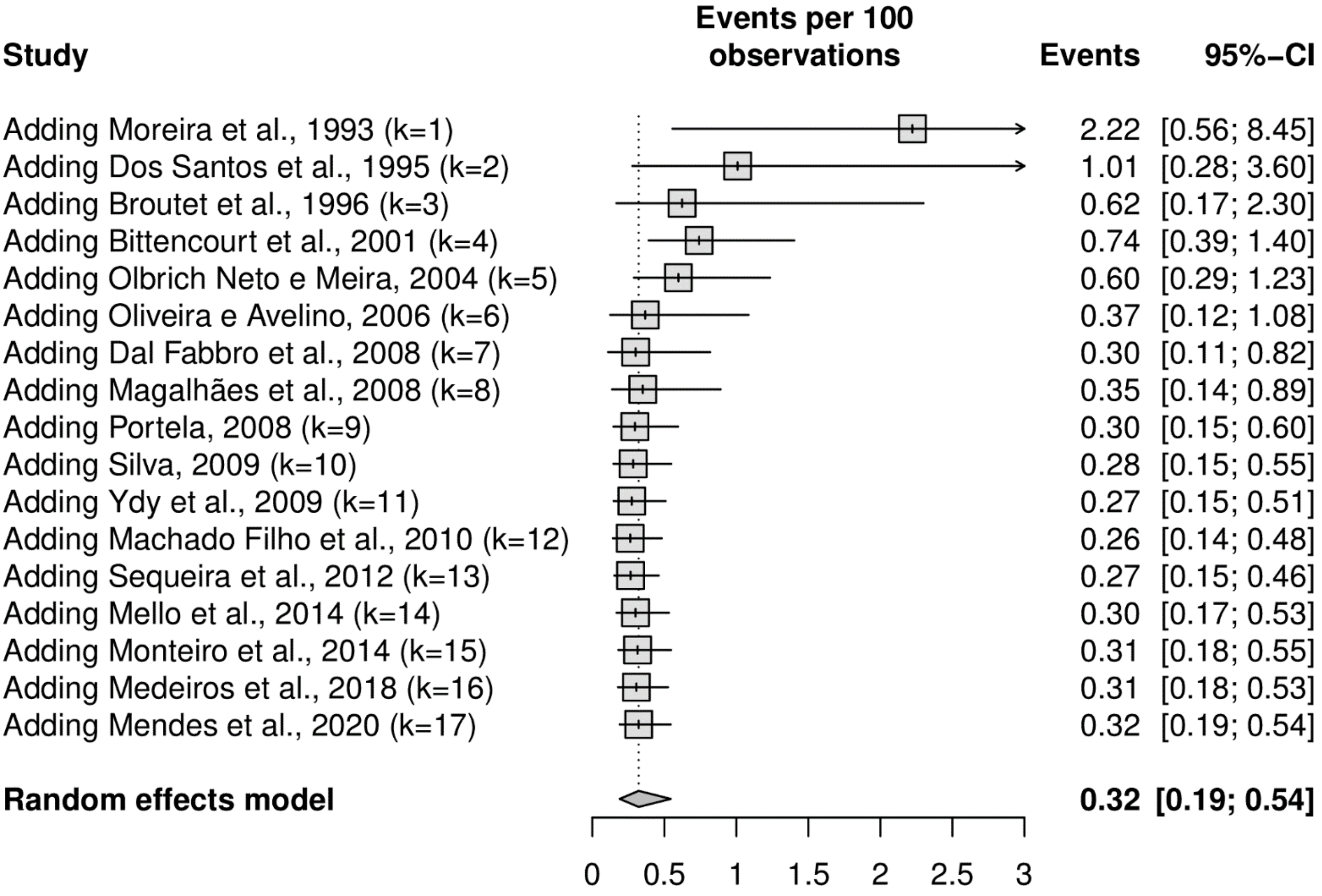

Forest plot of cumulative prevalence of HTLV-1 infection in pregnant women (1993-2020).

Supplementary Figure S2

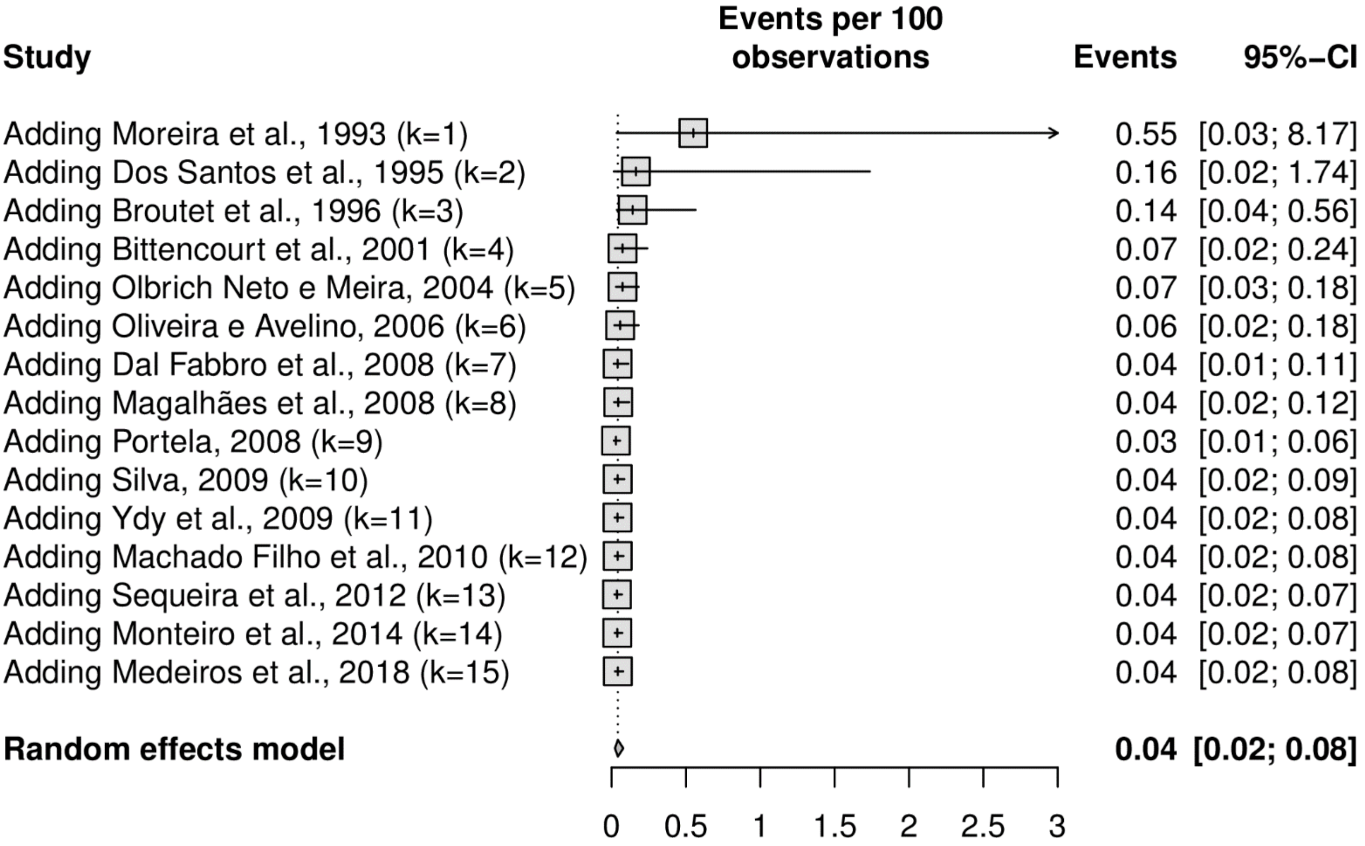

Forest plot of cumulative prevalence of HTLV-2 infection in pregnant women (1993-2018).

Supplementary Figure S3

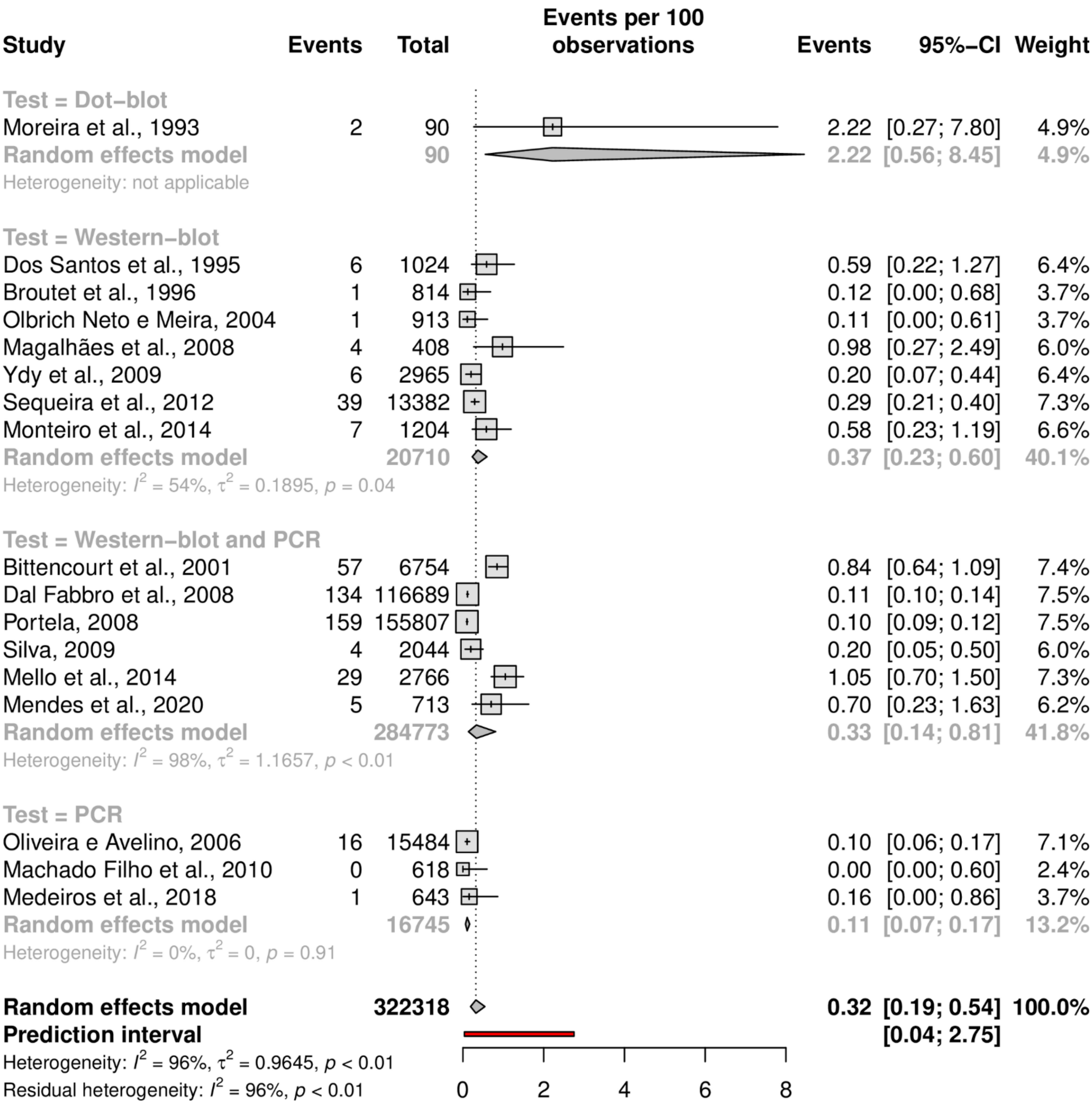

Forest plot of HTLV-1 infection in pregnant women by testing method (Western Blot, PCR or both).

Supplementary Figure S4

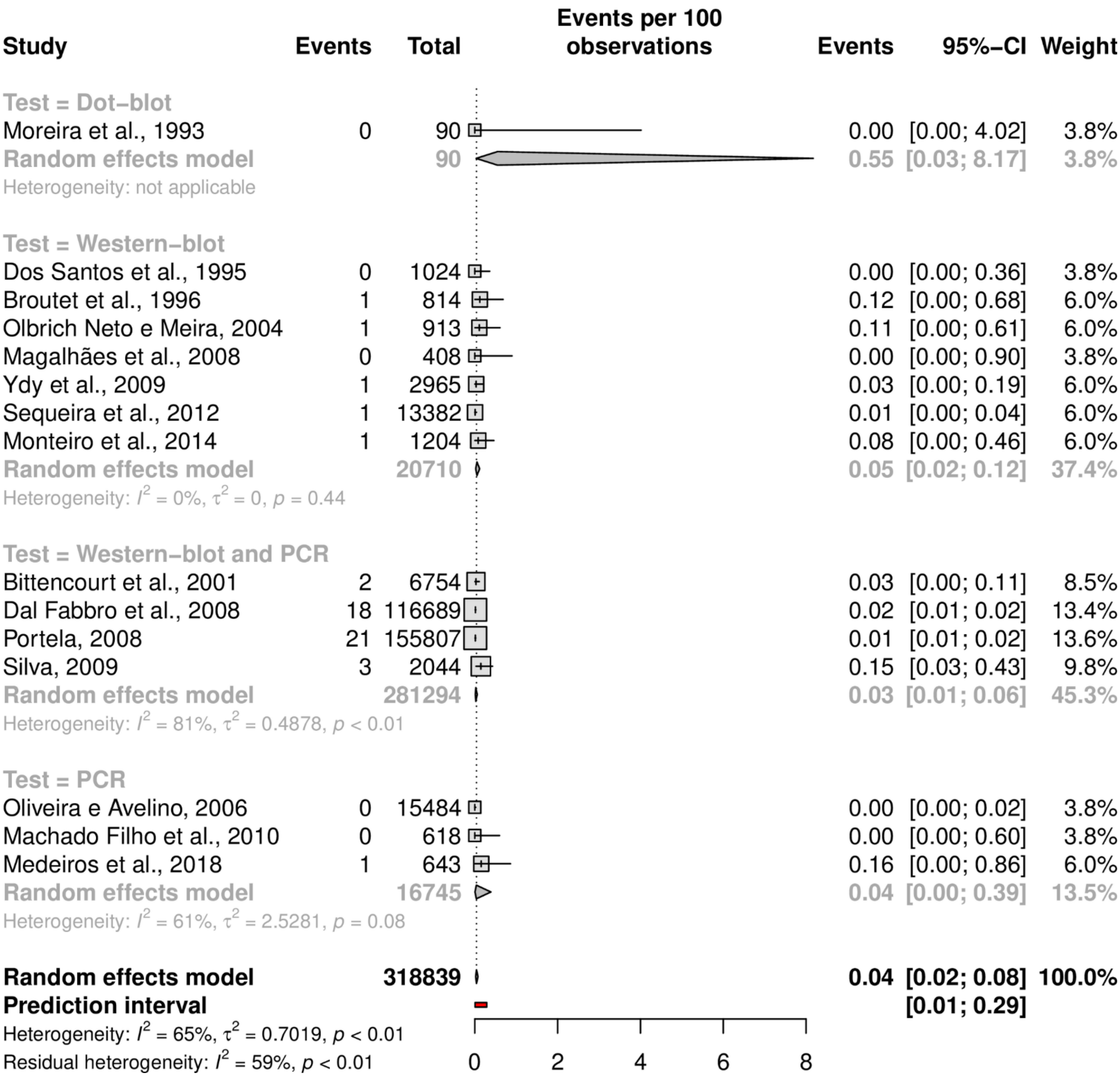

Forest plot of HTLV-2 infection in pregnant women by testing method (Western Blot, PCR or both).
